# Early Emotional Symptoms Predicting Carotid Atherosclerosis in Youth: Results From a Birth Cohort in Latin America

**DOI:** 10.1161/JAHA.118.011011

**Published:** 2019-01-17

**Authors:** Cristiano Tschiedel Belem da Silva, Maurício Scopel Hoffmann, Roberto Tofani Sant′Anna, Fernando César Wehrmeister, Helen Gonçalves, Isabel O. Oliveira, Paula Duarte Oliveira, Antônio Marcos Vargas da Silva, Christian Kieling, Gisele Gus Manfro, Ana M. B. Menezes

**Affiliations:** ^1^ School of Medicine Universidade do Vale do Rio dos Sinos São Leopoldo Rio Grande do Sul Brazil; ^2^ Universidade Federal de Santa Maria Santa Maria Rio Grande do Sul Brazil; ^3^ Instituto de Cardiologia Fundação Universitária de Cardiologia Clinical Research Center Porto Alegre Rio Grande do Sul Brazil; ^4^ Postgraduate Program in Epidemiology Federal University of Pelotas Rio Grande do Sul Brazil; ^5^ Department of Physiology and Pharmacology Federal University of Pelotas Capão do Leão Rio Grande do Sul Brazil; ^6^ Graduate Program in Functional Rehabilitation Federal University of Santa Maria Rio Grande do Sul Brazil; ^7^ Graduate Program in Psychiatry and Behavioral Sciences Federal University of Rio Grande do Sul Porto Alegre Rio Grande do Sul Brazil

**Keywords:** anxiety, atherosclerosis, carotid intima‐media thickness, depression, risk factor, Mental Health, Pediatrics, Risk Factors, Atherosclerosis, Vascular Disease

## Abstract

**Background:**

Emotional disorders are risk factors for atherosclerosis and consequent cardiovascular disease. However, it is not clear whether emotional symptoms (ESs) have direct effects on cardiovascular disease. The aim of the present study is to investigate the effects of early ESs on carotid atherosclerosis in young adults.

**Methods and Results:**

We tested the association between expression of ESs at 11 and 15 years of age and carotid intima‐media thickness at 18 years of age in the 1993 Pelotas Birth Cohort (N=5249, n=4336 with complete mental health data). ESs were assessed using the Strengths and Difficulties Questionnaire. Propensity score weighting procedure was run using generalized boosted regression model to adjust for potential confounding between exposure and outcome. We also tested whether traditional cardiovascular risk factors could mediate this relationship. Adjusted high expression of ESs, both at 11 and 15 years of age, led to mean increases in carotid intima‐media thickness of 1.84 and 2.58 μm, respectively, at 18 years of age (both *P*<0.001). Longitudinal effects of ESs on atherosclerosis were direct and not significantly mediated by traditional cardiovascular risk factors. Male sex at age 15 years significantly enhanced the effects of ESs on carotid intima‐media thickness at age 18 years (*P*<0.001 for interaction): although high expression of ESs led to mean increases of 1.14 μm in females (*P*<0.05), it led to mean increases of 5.83 μm in males (*P*<0.001).

**Conclusions:**

In this large birth cohort, expression of ESs in adolescence was longitudinally associated with a higher carotid intima‐media thickness in young adults. The association is direct and not mediated by traditional cardiovascular risk factors. Interactions by sex might have important implications for designing future interventions.

**Clinical Perspective**

**What Is New?**

- Adolescents with high emotional symptoms (ESs) have increased carotid intima‐media thickness in early adulthood.
- The effects of ESs are not mediated by traditional cardiovascular risk factors.
- Female sex buffers the effects of ESs on carotid intima‐media thickness.

**What Are the Clinical Implications?**

- Early interventions might be useful in mitigating the effects of ESs over cardiovascular health.
- Interventions tailored to male adolescents may have greater impact and should be prioritized.
- Referring adolescents experiencing ESs to mental health services shall be considered by physicians as a measure also promoting cardiovascular health.

## Introduction

Cardiovascular diseases (CVDs) are the leading cause of death worldwide. Strategies to decrease risk factors could prevent at least 80% of CVDs.^1^ Mental disorders (MDs) are associated with the development of CVD and are related to worse outcome once CVD is established.^2^ Multiple mechanisms link MDs, such as anxiety and depression, to CVD, including endothelial dysfunction,^3^ autonomic nervous system activation,^4^ elevated inflammatory activity,^5^ and lower adhesion to treatment and lifestyle changes in the context of established CVD.^6^ Atherosclerosis is the most frequent underlying cause of CVD. It is the consequence of a multifactorial process beginning in childhood. Interventions to reduce risk factors can lead to a greater reduction of CVD compared with target interventions adopted later in life.^7^ MDs are youth disorders and potentially contribute to atherosclerosis and CVD.^8^ Therefore, establishing a clear association between these conditions at an early age could lead to more effective interventions.

Carotid intima‐media thickness (cIMT) is a surrogate marker for the presence and progression of atherosclerosis.^9^ It is widely adopted because it can be simply, reproducibly, and noninvasively measurable. Exposure to traditional cardiovascular risk factors during childhood is correlated to increased cIMT.^8^ However, >60% of cIMT variance cannot be explained by traditional risk factors,^10^ which makes identifying novel risk factors an issue of paramount importance.

Adverse childhood experiences, for instance, are examples of candidate risk factors worth investigating further. They are related to greater blood pressure levels^11^ and CVD in adulthood.^12^ Recently, the American Heart Association published a scientific statement reviewing the mechanisms through which adversity could lead to CVD, one of which being poor mental health.^13^ It emphasized the paucity of longitudinal studies on the subject and the need to answer the question on how psychosocial risk factors lead to CVD.^14^

The Pelotas Birth Cohort is among the few cohorts in developing countries that follows individuals since their birth and provides data on child and adolescent general and mental health.^15^ In the present study, we analyzed the cohort of individuals born in 1993. We hypothesized that the presence of emotional symptoms (ESs) during childhood and adolescence would be associated with increased cIMT at early adulthood. Furthermore, our hypothesis was that traditional CVD risk factors, such as serum lipid markers, blood pressure, body mass index (BMI), physical activity, and serum blood glucose, would not significantly mediate the association.

## Methods

### Study Population

The data that support the findings of this study are available from the corresponding author on reasonable request.

In 1993, all mothers who delivered live‐born infants in all 5 city hospitals of Pelotas (Southern Brazil) were invited to join the study. A total of 5249 individuals agreed to participate, with only 16 subjects (0.3%) refusing participation. Written informed consent was obtained before entry from the primary caregiver and from all adolescents subsequently. This study was approved by the Institutional Review Board of the School of Medicine, Universidade Federal de Pelotas, Rio Grande do Sul (Brazil).

In the first visit, information on gestational age in weeks was estimated from the last menstrual period or using the Dubowitz method when the information on the last menstrual period was not available. Birth weight (pediatric scales [Filizola, Sao Paulo, Brazil], with a precision of 10 g), maternal skin color (white/nonwhite), maternal schooling (complete years), family income (in minimum wages), and smoking during pregnancy (yes/no) were also obtained. Subsequent follow‐up visits were conducted when children were 11, 15 and 18 years old. Response rates of 87.5%, 85.7%, and 81.3% were obtained when adolescents and young adults were 11, 15 and 18 years old, respectively.^16^, ^17^

### Measurements

#### Covariates

Several measures were collected at follow‐up visits: BMI (World Health Organization *z* score) categorized as underweight/normal (≤1 *z* score), overweight (>1 and ≤2 *z* score), or obese (>2 *z* score)^18^; sexual maturation (Tanner's stages of maturation)^19^, ^20^; parental history of hypertension and diabetes mellitus (yes/no); and smoking status (never/former/current).

Interviewers underwent standardization testing to ascertain repeatability of weight and height assessments before data collection and every 2 months afterward.

### Psychiatric Measures

ESs were assessed by trained interviewers using the validated Brazilian Portuguese version of the Strengths and Difficulties Questionnaire (SDQ).^21^, ^22^ A previous study of our group showed that it can be a useful tool to evaluate ESs when diagnoses provided by mental health clinicians are not feasible.^23^ The SDQ is a screening instrument for mental health problems consisting of 25 items, divided into 5 subscales (5 items each): ESs, hyperactive behavior, conduct problems, prosocial behavior, and peer relationships. Mental health symptoms were informed by primary caregivers. Adolescents were regarded as high in ESs at 11 or 15 years of age when their ES SDQ subscales (sum score of the 5 items) were 2 SDs above the mean of the sample. The same criterion was applied to the SDQ hyperactivity and conduct subscales to consider adolescents high in hyperactivity behavior and conduct problems (sensitivity analysis, see below). Dichotomizing into 2 SDQ groups enabled computation of propensity scores (PSs); establishing the cutoff of 2 SDs maximized specificity and positive predictive value for depressive and generalized anxiety disorders.^24^

Childhood adversity was assessed at 15 years old and consisted of a confidential questionnaire, including 7 dichotomous items filled by adolescents. Questions addressed lifetime physical, sexual, or emotional abuse and child neglect. Individuals were classified according to the presence and degree of maltreatment: no maltreatment (no positive answer), probable maltreatment (1 positive answer), and severe maltreatment (≥2 positive answers).^25^

When children were 11 years old, their mothers were assessed using the Brazilian Portuguese validated version of the Self‐Report Questionnaire.^26^ The Self‐Report Questionnaire estimates the occurrence of nonpsychotic psychiatric disorders over the former month, especially anxiety disorders and depression.^27^

### cIMT and Cardiovascular Risk Assessment

cIMT was measured at 18 years old: the posterior walls of the right and left common carotid arteries in longitudinal planes were assessed using ultrasound B‐mode imaging (Xario, Premium Compact, Toshiba), equipped with a 7.5‐MHz (5.0–11.0‐MHz) linear array transducer with 4‐cm deep and gain settings optimized to image quality. Subjects were positioned in the supine position with the head tilted 45° in the opposite direction to the examined carotid to have their scans done by a trained examiner. The examiner was blind and thus unaware of psychiatric symptoms presented by individuals. A section proximal to the carotid bulb of the common carotid artery was imaged in a moving scan with a duration of 8 s. Images were recorded in DICOM 3.0 format and analyzed using the Carotid Analyzer for Research software (Medical Imaging Applications, MIA‐LLC). The software automatically computed the mean value of 90 measurements (frames) taken in the 10‐mm‐long section located ≈10 mm to the carotid bulb.^28^

Blood pressure was computed as the mean of 2 measurements using an OMRON HEM 705CPINT digital upper arm device. Mean arterial pressure was calculated as follows: diastolic pressure+(systolic pressure−diastolic pressure)/3. Biochemical examinations were collected and comprised nonfasting blood: triglycerides (mg/dL), glycated hemoglobin (percentage), and plasma high‐ and low‐density lipoprotein cholesterol (mg/dL).

### Statistical Analyses

Because of the observational nature of the present study and to minimize confounding, we modeled the probabilities of developing high ESs at 11 and 15 years old using the PS weighting (PSW) method, which used generalized boosted modeling (GBM) to calculate PSs, in *twang* package in R.^29^ GBM has been made popular in the machine learning community as one of the latest prediction methods, allowing researchers to powerfully estimate exposure probability (PS) on the basis of many predicting covariates. It fits several models, both linear and nonlinear, using a regression tree and then merging predictions computed by each model.^30^, ^31^ Regression trees do not require researchers to specify functional forms of variables (ie, they handle continuous, nominal, ordinal, and missing independent variables, as well as nonlinear and interaction effects).^32^ Covariables used to compute PS at 11 years old and PS at 15 years old using GBM were chosen considering previous work on ESs and CVD risk^2^, ^33^, ^34^ and can be found in the Figure. Number of interaction trees was set on 5000, shrinkage in 0.01 and level of interactions in 2, which were basically set to minimize prediction errors by means of subsampling strategies.^32^ Balance in covariables between groups (eg, high versus low ESs) was ascertained comparing standardized mean differences. To allow further multivariate analysis having cIMT at 18 years old as the outcome, thus retaining as many individuals for the main analyses as possible, we opted not to match individuals on the basis of their PS. Instead, we computed the inverse probability of exposure weight, including it as a parameter in regression analysis (PSW), weighting the comparison cases to estimate the average treatment effect on the treated. The PSW also balances groups in terms of missingness of covariates, which is another advantage of this method.

**Figure 1:**
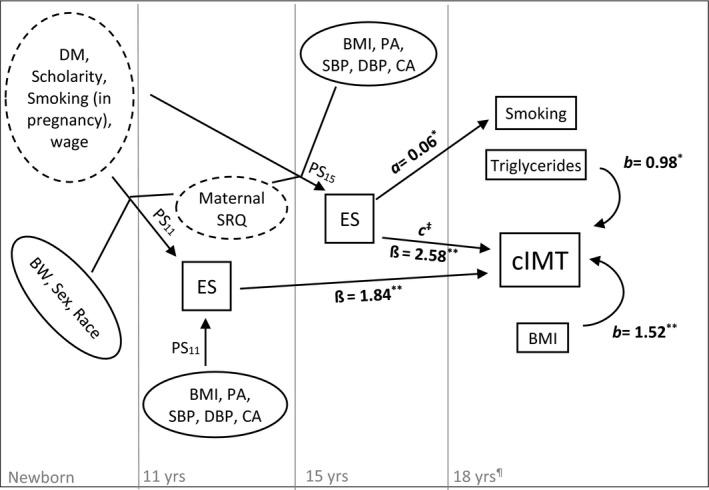
Timeline of data collection and diagram indicating direct and indirect paths across studied variables. Dashed oval: maternal/paternal variables; continuous ovals: variables directly assessed on adolescents at distinct waves. High‐density lipoprotein, low‐density lipoprotein, mean arterial pressure, and glycated hemoglobin assessed at 18 years old had no significant direct or indirect effects on carotid intima‐media thickness (cIMT). a=effect of exposure on the mediator; b=effect of the mediator on the outcome; c=direct effect of the exposure on the outcome. Estimates of mediated effects (ab) ranged between −0.10 and 0.09 (*P* range between 0.81 and 0.26). ^‡^Estimates of direct effects of emotional symptom (ESs) on cIMT were computed separately in mediation analyses and ranged between 2.3 and 2.6 (all *P*<0.05). BMI indicates body mass index; BW, birth weight; CA, childhood adversity; DBP, diastolic blood pressure; DM, diabetes mellitus; PA, physical activity; PS_11_, propensity score for ESs at 11 years old; PS_15_, propensity score for ESs at 15 years old; SBP, systolic blood pressure; SRQ, Self‐Reporting Questionnaire. *P<0.05, **P<0.01.

Because potential mediators could explain part of the associations between ESs and cIMT, we performed mediation analyses using structural equation modeling, taking PSW into account and using *lavaan* package in R.^35^ On the basis of previous research,^36^ triglycerides, low‐density lipoprotein, high‐density lipoprotein, glycated hemoglobin, smoking, mean arterial pressure, and BMI were tested as mediators, each in a separate analysis. To ensure ignorability in the associations of ESs with mediators and cIMT at 18 years old, we also included PSW scores in the structural equation models using *lavaan.survey* package in R.^37^

## Results

### Participant Characteristics

Data on the sample with complete mental health data corresponding to 2 waves, when individuals were 11 (n=4423) and 15 (n=4336) years old, of the Pelotas Birth Cohort were analyzed to generate separate PSW. Baseline characteristics of both waves, before and after weighting for the PS, are outlined in Tables 1 and 2. Most confounders were balanced (standardized mean difference, <0.2) between weighted groups, except for minor differences in family income at 15 years old, favoring the group with low ESs. Figures S1 and S2 provide the log likelihood explained by each input variable provided by GBM. Childhood adversity exerted negligible influence on the PS for ESs both at 11 and 15 years old (Figures S1 and S2). All adolescents included in the groups with high ESs scored >9 of 10 items that compose the SDQ emotional subscale.

**Table 1:** Unweighted and Weighted Means for Covariates and SMDs According to SDQ Group at 11 Years Old: The Pelotas Birth Cohort

Variable	Unweighted			Weighted		
	Emotional Symptoms		SMD	Emotional Symptoms		SMD
	Low (n=4244)	High (n=179)		Low (n=4244)	High (n=179)	
Female sex, %	50	58	0.16	56	58	0.03
Ethnicity (black), %	13	20	0.21	20	20	0.06
Maternal smoking during pregnancy, %	33	40	0.13	42	40	0.04
Diabetes mellitus (any parent), %	8	10	0.07	9	10	0.03
SBP, mm Hg	102	102	0.04	102	102	0.01
DBP, mm Hg	63	63	0.00	63	63	0.02
Birth weight, kg	3181	3033	0.27	3044	3033	0.02
Family income (minimum wages)	4.3	2.8	0.65	2.93	2.74	0.08
Maternal scholarity, y	6.7	5.2	0.62	5.3	5.2	0.05
Physical activity, min/wk	416	413	0.01	401	413	0.02
BMI, kg/m^2^						
Low/normal, %	69	74	0.11	74	74	0.01
Overweight, %	20	15	0.14	17	15	0.06
Obese, %	11	11	0.02	9	11	0.06
Childhood adversity^a^	0.23	0.22	0.01	0.22	0.22	0.00
Maternal SRQ^a^	7	11	1.10	11	11	0.12

BMI indicates body mass index; DBP, diastolic blood pressure; SBP, systolic blood pressure; SDQ, Strengths and Difficulties Questionnaire; SMD, standardized mean difference; SRQ, Self‐Reporting Questionnaire.

a Total scores.

**Table 2:** Unweighted and Weighted Means for Covariates and SMDs According to SDQ Group at 15 Years Old: The Pelotas Birth Cohort

Variable	Unweighted			Weighted		
	Emotional Symptoms		SMD	Emotional Symptoms		SMD
	Low (n=4197)	High (n=139)		Low (n=4197)	High (n=139)	
Female sex, %	50	73	0.50	68	73	0.11
Ethnicity (black), %	14	27	0.29	22	27	0.10
Maternal smoking during pregnancy, %	33	32	0.03	38	32	0.14
Diabetes mellitus (any parent), %	8	9	0.05	9	9	0.02
SBP, mm Hg	122	120	0.12	121	120	0.06
DBP, mm Hg	77	78	0.04	78	78	0.01
Birth weight, kg	3180	3094	0.16	3116	3094	0.04
Family income (minimum wages)	4.3	2.5	0.85	2.9	2.5	0.20
Maternal scholarity, y	6.8	4.9	0.76	5.2	4.9	0.10
Physical activity, min/wk	446	413	0.07	427	413	0.03
BMI, kg/m^2^						
Low/normal, %	68	71	0.06	67	71	0.09
Overweight, %	18	16	0.06	18	16	0.05
Obese, %	8	5	0.15	7	5	0.11
Tanner stage	4.0	4.0	0.04	4.0	4.0	0.03
Childhood adversity^a^	0.23	0.21	0.06	0.23	0.20	0.05
Maternal SRQ	7.0	10.3	0.70	9.8	10.3	0.11

BMI indicates body mass index; DBP, diastolic blood pressure; SBP, systolic blood pressure; SDQ, Strengths and Difficulties Questionnaire; SMD, standardized mean difference; SRQ, Self‐Reporting Questionnaire.

a Total score.

### Effects of ESs on cIMT

Individuals with complete data on ESs and cIMT at 11 (n=3068) and 15 (n=3136) years old were analyzed. After weighting for the PS, high levels of ESs, both at 11 and 15 years old, led to mean increases of 1.84 and 2.58 μm, respectively, in cIMT at 18 years old (both *P*<0.001). Table 3 depicts the results for linear models weighted for the PS. Being female (interaction *P*<0.001) buffered the effects of high levels of ESs at 15 years old over cIMT at 18 years old. Thus, we stratified analyses by sex and computed the effects of high ESs at 15 years old over cIMT at 18 years old, weighting for PS generated for each sex (using the same covariables as for PS at 15 years old). High ESs at 15 years old predicted a mean increase of 1.14 μm in cIMT in females (*P*<0.05), whereas it predicted a mean increase of 5.83 μm in males (*P*<0.001). Moreover, being a smoker at age 18 years increased the effects of ESs over cIMT (interaction *P*<0.001). Interestingly, individuals with higher pubertal development at 15 years old had more pronounced effects of ESs on subsequent cIMT (interaction *P*<0.01).

**Table 3:** PS Weighted Linear Regression Predicting cIMT at 18 Years Old: The Pelotas Birth Cohort

Age/Predictor	β	SEM	*t* Value	*P* Value
Aged 11 y (n=3068)				
High ESs	1.84	0.63	4.07	<0.001
Female sex	−2.02	0.62	3.26	<0.01
High ESs×female sex	−1.07	0.85	1.26	0.21
Aged 15 y (n=3136)				
High ESs	2.58	0.39	6.70	<0.001
Female sex	−2.33	0.59	3.96	<0.001
High ESs×female sex	−3.91	0.81	4.85	<0.001
High ESs×smoking	5.06	1.04	4.89	<0.001
High ESs×Tanner stages	0.89	0.28	3.12	<0.01

B indicates micrometers; cIMT, carotid intima‐media thickness; ES, emotional symptom; PS, propensity score.

### Mediation Analyses

Herein, we tested the possibility that traditional risk factors associated with increased atherosclerosis could be mediating the effects between ESs and cIMT. For this purpose, we used the structural equation modeling framework and tested, in separate models, each variable weighted for the PSW as a possible mediator between ESs at 15 years old and cIMT at 18 years old. As can be seen in the Figure, smoking, glycated hemoglobin, triglycerides, low‐density lipoprotein, high‐density lipoprotein, BMI, and mean arterial pressure assessed at 18 years old were not significant mediators between ESs and cIMT.

### Sensitivity Analysis

To test the specificity of the relationship between ESs and cIMT, we also computed PS predicting high hyperactivity behavior and high conduct problems at 15 years old. For this purpose, we included the same variables as in the PS for high ESs. Group differences (low versus high hyperactivity behavior and low versus high conduct problems) were negligible after balancing for the PSW, as can be seen in Tables S1 and S2, respectively. More important, after balancing for the PSW, high hyperactivity behavior at 15 years old was not a significant predictor of cIMT at 18 years old (β=0.19, SEM=0.54, *t*=0.34, *P*=0.73). Similarly, high conduct problems at 15 years old did not significantly predict cIMT at 18 years old (β=0.32, SEM=0.42, *t*=0.76, *P*=0.49). Also, repeating analyses with emotional SDQ at 11 and 15 years old as continuous variables predicting cIMT at 18 years old did not change the pattern of results (data available on request).

## Discussion

This study shows that ESs presented in 2 distinct moments of adolescence predicted higher levels of cIMT at young adulthood. The effects of ESs on cIMT were not mediated by traditional cardiovascular risk factors. The association was more pronounced in men than women and was significantly potentiated by current smoking status. Remarkably, our findings replicate, in a birth cohort of a developing country, prospective effects of ESs over carotid atherosclerosis. Such effects were specific to the expression of ESs, once the association did not hold true for those with hyperactivity behavior, nor for those with conduct problems.

Two previous large population‐based studies showed associations between depressive and anxiety symptoms in children and young adults and subsequent premature mortality by ischemic heart disease.^38^, ^39^ Both studies relied on robust adjustment for potential confounders, but putative mechanisms were not investigated. Inflammation, oxidative stress, and platelet, autonomic, and endothelial dysfunction were speculated among possible pathophysiological mediators.

cIMT is a marker of systemic atherosclerosis and involves both inflammation and oxidative stress, being associated with autonomic and endothelial dysfunctions.^40^ Anxiety symptoms have been prospectively associated with cIMT increases after 5 years of follow‐up, independently of traditional cardiovascular risk factors, in the EVA (*Etude sur le Vieillissiment Artériel*) study, which was conducted with nearly 400 middle‐aged adults.^33^ However, 2 epidemiological cohort studies, the Cardiovascular Risk in Young Finns Study and the BLSA (Baltimore Longitudinal Study of Aging),^41^, ^42^ failed to find the same association. Inconsistent results might be explained by different intervals between psychiatric and cIMT assessments, distinct instruments for measuring ESs, clinical comorbidities, and samples drawn from populations of distinct ages. In contrast, our study was larger and applied PSW, a robust statistical technique that provided more power than those of the above‐mentioned cohorts. Indeed, also applying PS method and assessing a larger sample, a robust association between generalized anxiety disorder and heart rate variability has been found, which is another proposed mechanism linking ESs to CVD.^43^

A meta‐analysis of prospective studies has shown that an absolute difference of 100 μm in cIMT increases by 10% to 15% and by 13% to 18% the risk of myocardial infarction and stroke, respectively.^44^ Therefore, taken alone, the 1.84‐ and 2.58‐μm cIMT increases found herein should not raise major concerns. Nevertheless, the above‐mentioned review included samples with mean ages ranging between 50 and 73 years old, which sharply contrasts with our sample of youngsters. Furthermore, the mean follow‐up of included studies was only 5.5 years. Because it has been demonstrated that the relationship between cIMT and CVD is nonlinear in young individuals,^44^ extrapolations from older samples should be taken with extreme caution. Young adults are rarely under a physician's supervision and thus seldom medicated, which contributes for further carotid thickening once the process has started. Consistent with this view, our findings show that deleterious effects of ESs on cIMT increased between 11 and 15 years old. Indeed, ESs are chronic and recurrent and increasing effects on cIMT throughout young adulthood might lead to a much greater CVD impact than once expected.

Serum lipid markers, blood pressure, BMI, and blood glucose did not significantly mediate the association between ESs and cIMT. Although such adverse lifestyle behaviors are usually associated with negative emotional states,^13^ shared genetic mechanisms may better explain the phenomenon. Previous research has shown that healthy adolescents with parental history of major depressive disorder presented increased aortic stiffness, blood pressure, and insulin resistance compared with controls with no parental history of major depressive disorder.^34^ Moreover, the offspring of parents with CVD are more likely to have presented an episode of major depressive disorder than controls with no familial history of CVD.^45^ Consistent with a pleiotropic effect, our results show that parental history of elevated blood pressure and diabetes mellitus, along with mental health symptoms, exerts a robust influence in the PS for ESs in their siblings. Again, PSW balanced for such a complex interplay between CVD and ESs as mutual risk factors, which strengthens the effects shown herein.

Three additional findings deserve further comment. First, we found a significant interaction between ESs and smoking in predicting higher cIMT. A possibility is that both ESs and smoking share a common pathway leading to atherosclerosis (eg, systemic inflammation).^46^, ^47^ Second, the effects of ESs on cIMT seemed to be more pronounced in male than in female adolescents. Certain genetic variants, such as the phosphodiesterase 4D gene, are specifically associated with higher cIMT levels in males, but not in females.^48^ Interestingly, the phosphodiesterase 4 isoenzyme family is widely expressed within the central nervous system, and the disruption of its activities is associated with mood and memory disturbances.^49^ Thus, a conjectural explanation would be that males with such genetic variants would be susceptible to both ESs and atherosclerosis. Third, there was a significant ES by pubertal stage interaction on cIMT, irrespectively of sex. Although all individuals from our sample were born in the same year, this might indicate that not only chronological age, but also fluctuations within hormonal environment, may play a role in the pathophysiological characteristics of early atherosclerosis.

Our study needs to be understood in light of its limitations. First, this is an observational study and causal pathways might have confounding factors. However, using GBM within the PSW procedure, along with increasing effect size with age and specificity of ES effects on cIMT, altogether can minimize the influence of confounding factors and argue for effect directionality. Second, we used SQD as a marker of MDs. Although it is an established screening method for MDs, it does not replace validated diagnostic interviews. This could explain differences between other studies and ours. However, when compared with diagnostic measures assessing depression and generalized anxiety disorder, an equivalent cutoff has been shown to present optimal positive‐predictive (100%), specificity (100%), and negative‐predictive (80%) values.^24^ Therefore, the most likely was that adolescents with more severe symptoms of depression and anxiety were captured in our sample and that this may have decreased noise for statistically testing our hypotheses.

Our findings are consistent with a role of ESs on the pathophysiological characteristics of early atherosclerosis. Preliminary studies support the efficacy of psychological interventions to ameliorate cardiovascular outcomes in adults.^50^ However, randomized, double‐blind, placebo‐controlled studies that evaluate the utility of early interventions specifically designed to mitigate the effects of ESs in childhood and adolescence and their effects in atherosclerosis are still lacking.

## Sources of Funding

The cohort received funding from the following agencies: Wellcome Trust (No. 086974/Z/08/Z), International Development Research Center, World Health Organization, Overseas Development Administration of the United Kingdom, European Union, Brazilian National Support Program for Centers of Excellence (PRONEX), Brazilian National Council for Scientific and Technological Development (CNPq), Science and Technology Department (DECIT) of the Brazilian Ministry of Health, Research Support Foundation of the State of Rio Grande do Sul (FAPERGS), and Brazilian Association for Collective Health (ABRASCO). None of these organizations influenced the study design or collection, analysis, and interpretation of data; the writing of the report; or the decision to submit the manuscript for publication.

## Disclosures

None.

## Supporting information


**Table S1.** Unweighted and Weighted Means for Covariates and Standardized Mean Difserences (SMD) According to SDQ Group at 15 Years‐old: The Pelotas Birth Cohort
**Table S2.** Unweighted and Weighted Means for Covariates and Standardized Mean Differences (SMD) According to SDQ Group at 15 Years‐Old: The Pelotas Birth Cohort
**Figure S1.** Relative influence of each variable on the propensity score for high emotional symptoms at 11 years‐old.
**Figure S2.** Relative influence of each variable on the propensity score for high emotional symptoms at 15 years‐old.

## References

[1] Authors/Task Force Members , Piepoli MF , Hoes AW , Agewall S , Albus C , Brotons C , Catapano AL , Cooney M‐T , Corrà U , Cosyns B , Deaton C , Graham I , Hall MS , Hobbs FDR , Løchen M‐L , Löllgen H , Marques‐Vidal P , Perk J , Prescott E , Redon J , Richter DJ , Sattar N , Smulders Y , Tiberi M , Bart van der Worp H , van Dis I , Verschuren WMM . 2016 European guidelines on cardiovascular disease prevention in clinical practice: the Sixth Joint Task Force of the European Society of Cardiology and Other Societies on Cardiovascular Disease Prevention in Clinical Practice (constituted by representatives of 10 societies and by invited experts): developed with the special contribution of the European Association for Cardiovascular Prevention & Rehabilitation (EACPR). Atherosclerosis. 2016;252:207–274. 27664503 10.1016/j.atherosclerosis.2016.05.037

[2] Liu K , Daviglus ML , Loria CM , Colangelo LA , Spring B , Moller AC , Lloyd‐Jones DM . Healthy lifestyle through young adulthood and the presence of low cardiovascular disease risk profile in middle age: the Coronary Artery Risk Development in (Young) Adults (CARDIA) study. Circulation. 2012;125:996–1004. 22291127 10.1161/CIRCULATIONAHA.111.060681 PMC3353808

[3] Cooper DC , Tomfohr LM , Milic MS , Natarajan L , Bardwell WA , Ziegler MG , Dimsdale JE . Depressed mood and flow‐mediated dilation: a systematic review and meta‐analysis. Psychosom Med. 2011;73:360–369. 21636660 10.1097/PSY.0b013e31821db79a PMC4266566

[4] Carney RM , Freedland KE , Veith RC , Cryer PE , Skala JA , Lynch T , Jaffe AS . Major depression, heart rate, and plasma norepinephrine in patients with coronary heart disease. Biol Psychiatry. 1999;45:458–463. 10071718 10.1016/s0006-3223(98)00049-3

[5] Carney RM , Freedland KE . Depression and coronary heart disease. Nat Rev Cardiol. 2017;14:145–155. 27853162 10.1038/nrcardio.2016.181

[6] Gehi A , Haas D , Pipkin S , Whooley MA . Depression and medication adherence in outpatients with coronary heart disease: findings from the Heart and Soul Study. Arch Intern Med. 2005;165:2508–2513. 16314548 10.1001/archinte.165.21.2508 PMC2776695

[7] Ference BA , Yoo W , Alesh I , Mahajan N , Mirowska KK , Mewada A , Kahn J , Afonso L , Williams KA , Flack JM . Effect of long‐term exposure to lower low‐density lipoprotein cholesterol beginning early in life on the risk of coronary heart disease: a Mendelian randomization analysis. J Am Coll Cardiol. 2012;60:2631–2639. 23083789 10.1016/j.jacc.2012.09.017

[8] Lorenz MW , von Kegler S , Steinmetz H , Markus HS , Sitzer M . Carotid intima‐media thickening indicates a higher vascular risk across a wide age range: prospective data from the Carotid Atherosclerosis Progression Study (CAPS). Stroke. 2006;37:87–92. 16339465 10.1161/01.STR.0000196964.24024.ea

[9] Carpenter M , Sinclair H , Kunadian V . Carotid intima media thickness and its utility as a predictor of cardiovascular disease: a review of evidence. Cardiol Rev. 2016;24:70–75. 26825762 10.1097/CRD.0000000000000077

[10] Santos IS , Alencar AP , Rundek T , Goulart AC , Barreto SM , Pereira AC , Benseñor IM , Lotufo PA . Low impact of traditional risk factors on carotid intima‐media thickness: the ELSA‐Brasil cohort. Arterioscler Thromb Vasc Biol. 2015;35:2054–2059. 26183615 10.1161/ATVBAHA.115.305765

[11] Su S , Wang X , Pollock JS , Treiber FA , Xu X , Snieder H , McCall WV , Stefanek M , Harshfield GA . Adverse childhood experiences and blood pressure trajectories from childhood to young adulthood: the Georgia Stress and Heart Study. Circulation. 2015;131:1674–1681. 25858196 10.1161/CIRCULATIONAHA.114.013104 PMC4430378

[12] Basu A , McLaughlin KA , Misra S , Koenen KC . Childhood maltreatment and health impact: the examples of cardiovascular disease and type 2 diabetes mellitus in adults. Clin Psychol Sci Pract. 2017;24:125–139. 10.1111/cpsp.12191 PMC5578408 28867878

[13] Suglia SF , Koenen KC , Boynton‐Jarrett R , Chan PS , Clark CJ , Danese A , Faith MS , Goldstein BI , Hayman LL , Isasi CR , Pratt CA , Slopen N , Sumner JA , Turer A , Turer CB , Zachariah JP . Childhood and adolescent adversity and cardiometabolic outcomes: a scientific statement from the American Heart Association. Circulation. 2018;137:e15–e28. 29254928 10.1161/CIR.0000000000000536 PMC7792566

[14] Havranek EP , Mujahid MS , Barr DA , Blair IV , Cohen MS , Cruz‐Flores S , Davey‐Smith G , Dennison‐Himmelfarb CR , Lauer MS , Lockwood DW , Rosal M , Yancy CW . Social determinants of risk and outcomes for cardiovascular disease: a scientific statement from the American Heart Association. Circulation. 2015;132:873–898. 26240271 10.1161/CIR.0000000000000228

[15] Rocha TB‐M , Hutz MH , Salatino‐Oliveira A , Genro JP , Polanczyk GV , Sato JR , Wehrmeister FC , Barros FC , Menezes AMB , Rohde LA , Anselmi L , Kieling C . Gene‐environment interaction in youth depression: replication of the 5‐HTTLPR moderation in a diverse setting. Am J Psychiatry. 2015;172:978–985. 26315979 10.1176/appi.ajp.2015.14070896

[16] Victora CG , Barros FC , Tomasi E , Menezes AM , Horta BL , Weiderpass E , Cesar JA , Costa JSD , Olinto MT , Halpern R , Garcia M del M , Vaughan JP . Tendências e diferenciais na saúde materno‐infantil: delineamento e metodologia das coortes de 1982 e 1993 de mães e crianças de Pelotas, Rio Grande do Sul. Cad Saúde Pública. 1996;12:S7–S14.

[17] Gonçalves H , Assunção MC , Wehrmeister FC , Oliveira IO , Barros FC , Victora CG , Hallal PC , Menezes AM . Cohort profile update: the 1993 Pelotas (Brazil) birth cohort follow‐up visits in adolescence. Int J Epidemiol. 2014;43:1082–1088. 24729426 10.1093/ije/dyu077 PMC4121560

[18] de Onis M , Onyango AW , Borghi E , Siyam A , Nishida C , Siekmann J . Development of a WHO growth reference for school‐aged children and adolescents. Bull World Health Organ. 2007;85:660–667. 18026621 10.2471/BLT.07.043497 PMC2636412

[19] Marshall WA , Tanner JM . Variations in pattern of pubertal changes in girls. Arch Dis Child. 1969;44:291–303. 5785179 10.1136/adc.44.235.291 PMC2020314

[20] Marshall WA , Tanner JM . Variations in the pattern of pubertal changes in boys. Arch Dis Child. 1970;45:13–23. 5440182 10.1136/adc.45.239.13 PMC2020414

[21] Goodman R . The strengths and difficulties questionnaire: a research note. J Child Psychol Psychiatry. 1997;38:581–586. 9255702 10.1111/j.1469-7610.1997.tb01545.x

[22] Fleitlich‐Bilyk B , Goodman R . Prevalence of child and adolescent psychiatric disorders in southeast Brazil. J Am Acad Child Adolesc Psychiatry. 2004;43:727–734. 15167089 10.1097/01.chi.0000120021.14101.ca

[23] Anselmi L , Fleitlich‐Bilyk B , Menezes AMB , Araújo CL , Rohde LA . Prevalence of psychiatric disorders in a Brazilian birth cohort of 11‐year‐olds. Soc Psychiatry Psychiatr Epidemiol. 2010;45:135–142. 19381426 10.1007/s00127-009-0052-2

[24] Silva TBF , Osório FL , Loureiro SR . SDQ: discriminative validity and diagnostic potential. Front Psychol. 2015;6:811. 26113840 10.3389/fpsyg.2015.00811 PMC4462033

[25] Caspi A , McClay J , Moffitt TE , Mill J , Martin J , Craig IW , Taylor A , Poulton R . Role of genotype in the cycle of violence in maltreated children. Science. 2002;297:851–854. 12161658 10.1126/science.1072290

[26] Mari JJ , Williams P . A validity study of a psychiatric screening questionnaire (SRQ‐20) in primary care in the city of Sao Paulo. Br J Psychiatry. 1986;148:23–26. 3955316 10.1192/bjp.148.1.23

[27] Harding TW , De Arango V , Baltazar J , Climent CE , Ibrahim HHA , Ladrido‐Ignacio L , Wig NN . Mental disorders in primary health care: a study of their frequency and diagnosis in four developing countries. Psychol Med. 1980;10:231. 7384326 10.1017/s0033291700043993

[28] Menezes AMB , da Silva CTB , Wehrmeister FC , Oliveira PD , Oliveira IO , Gonçalves H , Assunção MCF , de Castro Justo F , Barros FC . Adiposity during adolescence and carotid intima‐media thickness in adulthood: results from the 1993 Pelotas Birth Cohort. Atherosclerosis. 2016;255:25–30. 27816805 10.1016/j.atherosclerosis.2016.10.026 PMC5152614

[29] Ridgeway G , McCaffrey D , Morral A , Griffin BA , Burgette L . twang: toolkit for weighting and analysis of nonequivalent groups [Internet]. 2017 https://CRAN.R-project.org/package=twang. Accessed April 4, 2017.

[30] Friedman JH , Meulman JJ . Multiple additive regression trees with application in epidemiology. Stat Med. 2003;22:1365–1381. 12704603 10.1002/sim.1501

[31] Guo S , Fraser MW . Propensity Score Analysis: Statistical Methods and Applications. 2nd ed Los Angeles, CA: SAGE; 2015.

[32] McCaffrey DF , Ridgeway G , Morral AR . Propensity score estimation with boosted regression for evaluating causal effects in observational studies. Psychol Methods. 2004;9:403–425. 15598095 10.1037/1082-989X.9.4.403

[33] Paterniti S , Zureik M , Ducimetière P , Touboul PJ , Fève JM , Alpérovitch A . Sustained anxiety and 4‐year progression of carotid atherosclerosis. Arterioscler Thromb Vasc Biol. 2001;21:136–141. 11145945 10.1161/01.atv.21.1.136

[34] Mannie ZN , Williams C , Diesch J , Steptoe A , Leeson P , Cowen PJ . Cardiovascular and metabolic risk profile in young people at familial risk of depression. Br J Psychiatry. 2013;203:18–23. 23703316 10.1192/bjp.bp.113.126987

[35] Rosseel Y . lavaan: an R package for structural equation modeling. J Stat Softw. 2012;48 http://www.jstatsoft.org/v48/i02/. Accessed February 11, 2018.

[36] Cao JJ , Arnold AM , Manolio TA , Polak JF , Psaty BM , Hirsch CH , Kuller LH , Cushman M . Association of carotid artery intima‐media thickness, plaques, and C‐reactive protein with future cardiovascular disease and all‐cause mortality: the Cardiovascular Health Study. Circulation. 2007;116:32–38. 17576871 10.1161/CIRCULATIONAHA.106.645606

[37] Oberski D . lavaan.survey: an R package for complex survey analysis of structural equation models. J Stat Softw. 2014;57 http://www.jstatsoft.org/v57/i01/. Accessed February 11, 2018.

[38] Huang K‐L , Su T‐P , Chen T‐J , Chou Y‐H , Bai Y‐M . Comorbidity of cardiovascular diseases with mood and anxiety disorder: a population based 4‐year study. Psychiatry Clin Neurosci. 2009;63:401–409. 19566773 10.1111/j.1440-1819.2009.01974.x

[39] Shah AJ , Veledar E , Hong Y , Bremner JD , Vaccarino V . Depression and history of attempted suicide as risk factors for heart disease mortality in young individuals. Arch Gen Psychiatry. 2011;68:1135–1142. 22065529 10.1001/archgenpsychiatry.2011.125 PMC3230326

[40] Halcox JPJ , Donald AE , Ellins E , Witte DR , Shipley MJ , Brunner EJ , Marmot MG , Deanfield JE . Endothelial function predicts progression of carotid intima‐media thickness. Circulation. 2009;119:1005–1012. 19204308 10.1161/CIRCULATIONAHA.108.765701

[41] Rice SC , Zonderman AB , Metter EJ , Najjar SS , Waldstein SR . Absence of relation between depressive symptoms and carotid intimal medial thickness in the Baltimore Longitudinal Study of Aging. Psychosom Med. 2009;71:70–76. 18842746 10.1097/PSY.0b013e3181865f73 PMC2628411

[42] Elovainio M , Keltikangas‐Järvinen L , Kivimäki M , Pulkki L , Puttonen S , Heponiemi T , Juonala M , Viikari JSA , Raitakari OT . Depressive symptoms and carotid artery intima‐media thickness in young adults: the Cardiovascular Risk in Young Finns Study. Psychosom Med. 2005;67:561–567. 16046368 10.1097/01.psy.0000170340.74035.23

[43] Kemp AH , Brunoni AR , Santos IS , Nunes MA , Dantas EM , Carvalho de Figueiredo R , Pereira AC , Ribeiro ALP , Mill JG , Andreão RV , Thayer JF , Benseñor IM , Lotufo PA . Effects of depression, anxiety, comorbidity, and antidepressants on resting‐state heart rate and its variability: an ELSA‐Brasil cohort baseline study. Am J Psychiatry. 2014;171:1328–1334. 25158141 10.1176/appi.ajp.2014.13121605

[44] Lorenz MW , Markus HS , Bots ML , Rosvall M , Sitzer M . Prediction of clinical cardiovascular events with carotid intima‐media thickness: a systematic review and meta‐analysis. Circulation. 2007;115:459–467. 17242284 10.1161/CIRCULATIONAHA.106.628875

[45] Rottenberg J , Yaroslavsky I , Carney RM , Freedland KE , George CJ , Baji I , Dochnal R , Gádoros J , Halas K , Kapornai K , Kiss E , Osváth V , Varga H , Vetró A , Kovacs M . The association between major depressive disorder in childhood and risk factors for cardiovascular disease in adolescence. Psychosom Med. 2014;76:122–127. 24470130 10.1097/PSY.0000000000000028 PMC4186704

[46] Durda P , Sabourin J , Lange EM , Nalls MA , Mychaleckyj JC , Jenny NS , Li J , Walston J , Harris TB , Psaty BM , Valdar W , Liu Y , Cushman M , Reiner AP , Tracy RP , Lange LA . Plasma levels of soluble interleukin‐2 receptor α: associations with clinical cardiovascular events and genome‐wide association scan. Arterioscler Thromb Vasc Biol. 2015;35:2246–2253. 26293465 10.1161/ATVBAHA.115.305289 PMC5395092

[47] Goldsmith DR , Rapaport MH , Miller BJ . A meta‐analysis of blood cytokine network alterations in psychiatric patients: comparisons between schizophrenia, bipolar disorder and depression. Mol Psychiatry. 2016;21:1696–1709. 26903267 10.1038/mp.2016.3 PMC6056174

[48] Liao Y‐C , Lin H‐F , Guo Y‐C , Yu M‐L , Liu C‐K , Juo S‐HH . Sex‐differential genetic effect of phosphodiesterase 4D (PDE4D) on carotid atherosclerosis. BMC Med Genet. 2010;11:93. 20540798 10.1186/1471-2350-11-93 PMC2895592

[49] Siuciak JA , McCarthy SA , Chapin DS , Martin AN . Behavioral and neurochemical characterization of mice deficient in the phosphodiesterase‐4B (PDE4B) enzyme. Psychopharmacology. 2008;197:115–126. 18060387 10.1007/s00213-007-1014-6

[50] Blumenthal JA , Sherwood A , Smith PJ , Watkins L , Mabe S , Kraus WE , Ingle K , Miller P , Hinderliter A . Enhancing cardiac rehabilitation with stress management training: a randomized clinical efficacy trial. Circulation. 2016;133:1341–1350. 27045127 10.1161/CIRCULATIONAHA.115.018926 PMC4824555

