# Supplementary material for: Early Emotional Symptoms Predicting Carotid Atherosclerosis in Youth: Results From a Birth Cohort in Latin America
Source: J Am Heart Assoc. 2019 Jan 17;8(2):e011011. doi: 10.1161/JAHA.118.011011 (PMC6497357; doi:10.1161/JAHA.118.011011)
Supplement: Supplementary file 1 — Table S1. Unweighted and Weighted Means for Covariates and Standardized Mean Difserences (SMD) According to SDQ Group at 15 Years‐old: The Pelotas Birth Cohort Table S2. Unweighted and Weighted Means for Covariates and Standardized Mean Differences (SMD) According to SDQ Group at 15 Years‐Old: The Pelotas Birth Cohort Figure S1. Relative influence of each variable on the propensity score for high emotional symptoms at 11 years‐old. Figure S2. Relative influence of each variable on the propensity score for high emotional symptoms at 15 years‐old. [file JAH3-8-e011011-s001.pdf]

SUPPLEMENTAL MATERIAL

Table S1. Unweighted and weighted means for covariates and standardized mean diferences (SMD) according to SDQ group at 15 years-old: The Pelotas Birth Cohort.

Variable	Unweighted			Weighted		
	Hyperactivity Behavior		SMD	Hyperactivity Behavior		SMD
	Low (n= 4,114)	High (n= 224)		Low (n= 4,114)	High (n= 224)	
Female sex (%)	52	37	.30	41	37	.08
Ethnicity (% black)	14	19	.14	18	19	.04
Maternal Smoking during Pregnancy (%)	33	44	.23	42	44	.05
Diabetes (% any parent)	8	8	.01	8	8	.01
SBP (mm/Hg)	122	121	.11	121	121	.02
DBP (mm/Hg)	77	76	.14	76	76	.003
Birth Weight (Kg)	3,180	3,121	.12	3,120	3,121	.001
Family Income (Minimum Wages)	4.3	3.4	.26	3.5	3.4	.03
Maternal Scholarity (yrs)	6.8	6.1	.20	6.2	6.1	.01
Physical Activity (min/wk)	440	544	.15	505	544	.06
BMI (Kg/m^2)						
Low/normal (%)	69	67	.04	68	67	.04
Overweight (%)	18	19	.04	18	19	.03
Obese (%)	8	9	.03	8	9	.05

Tanner Stage	4.0	4.2	.15	4.2	4.2	.02
Chilhood Adversity*	.23	.21	.07	.20	.21	.01
Maternal SRQ*	7.0	8.5	.34	8.4	8.5	.02

SBP = Systolic Blood Pressure; DBP = Diastolic Blood Pressure; BMI = Body Mass Index; SRQ = Self-reporting Questionnaire; *Total scores

Table S2. Unweighted and weighted means for covariates and standardized mean differences (SMD) according to SDQ group at 15 years-old: The Pelotas Birth Cohort.

Variable	Unweighted			Weighted		
	Conduct Problems		SMD	Conduct Problems		SMD
	Low (n= 4,079)	High (n= 259)		Low (n= 4,079)	High (n= 259)	
Female sex (%)	51	56	.10	52	56	.08
Ethnicity (% black)	14	22	.20	20	22	.05
Maternal Smoking during Pregnancy (%)	32	50	.35	46	50	.07
Diabetes (% any parent)	8	7	.04	9	7	.06
SBP (mm/Hg)	122	121	.08	121	121	.01
DBP (mm/Hg)	77	75	.19	75	75	.05
Birth Weight (Kg)	3,179	3,139	.08	3,145	3,139	.01
Family Income (Minimum Wages)	4.4	2.7	.66	2.9	2.7	.08
Maternal Scholarity (yrs)	6.8	5.3	.54	5.4	5.3	.03
Physical Activity (min/wk)	440	531	.15	508	531	.04
BMI (Kg/m^2)						
Low/normal (%)	68	71	.05	69	71	.03
Overweight (%)	18	16	.06	17	16	.03
Obese (%)	8	8	.02	8	8	.01

Tanner Stage	4.0	4.0	.00	4.0	4.0	.03
Chilhood Adversity*	.2	.2	.17	.2	.2	.05
Maternal SRQ*	7.0	9.4	.55	9.1	9.4	.06

SBP = Systolic Blood Pressure; DBP = Diastolic Blood Pressure; BMI = Body Mass Index; SRQ = Self-reporting Questionnaire; *Total scores

Figure S1. Relative Influence of Each Variable on the Propensity Score for High Emotional Symptoms at 11 years-old.

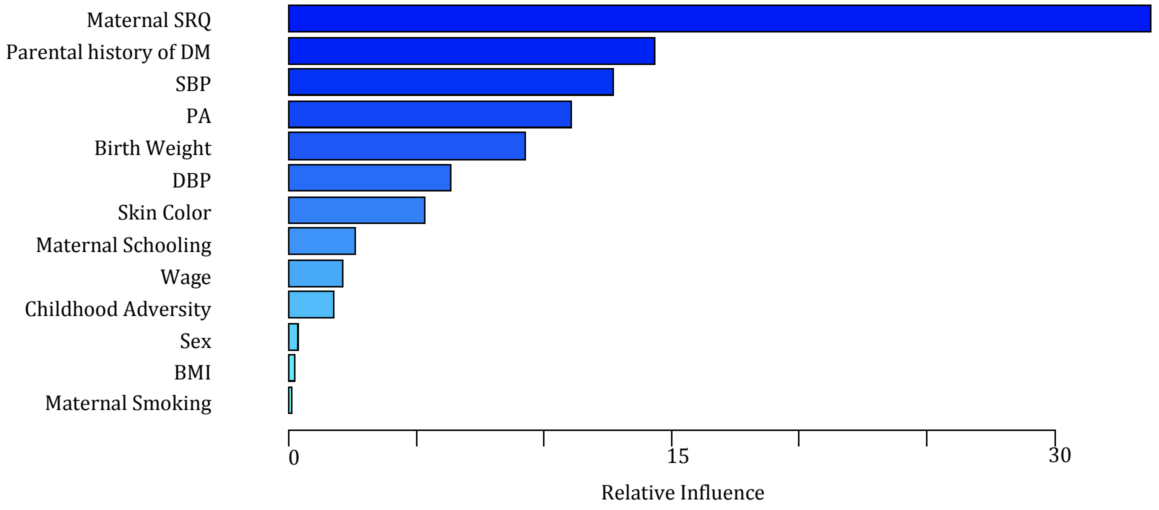

SRQ: Self-Report Questionnaire; DM: Diabetes Mellitus; SBP: Systolic Blood Pressure; PA: Physical Activity; DBP: Diastolic Blood Pressure; BMI: Body Mass Index.

Figure S2. Relative Influence of Each Variable on the Propensity Score for High Emotional Symptoms at 15 years-old.

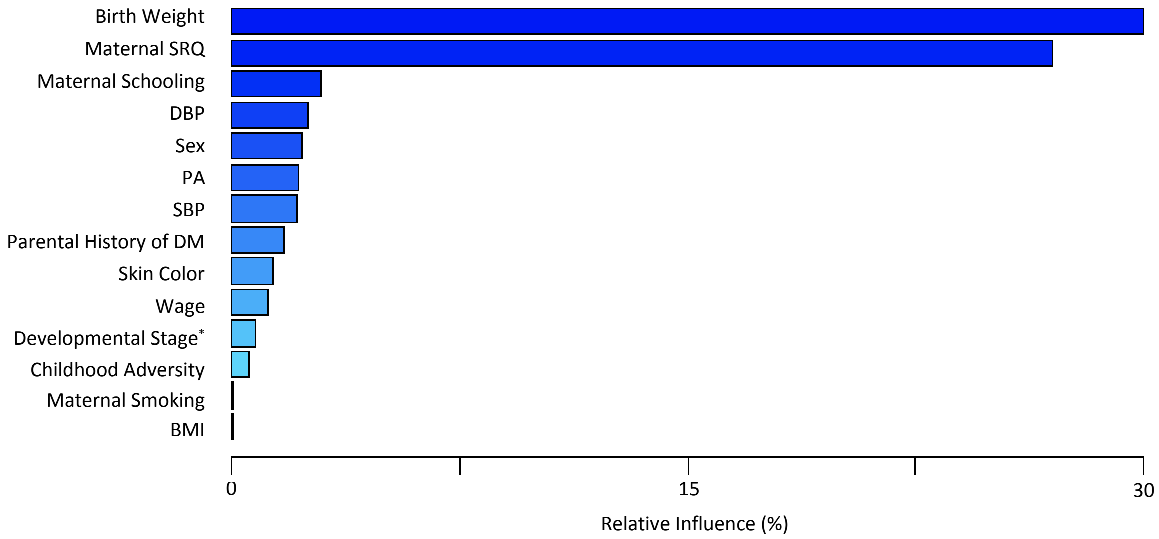

SRQ: Self-Report Questionnaire; DBP: Diastolic Blood Pressure; PA: Physical Activity; SBP: Systolic Blood Pressure; DM: Diabetes Mellitus; BMI: Body Mass Index. *Tanner Puberal Developmental Stage
